# Unlocking the Role of Metabolic Pathways in Brain Metastatic Disease

**DOI:** 10.3390/cells14100707

**Published:** 2025-05-13

**Authors:** Madalena Pinto, Sara Violante, Rita Cascão, Claudia C. Faria

**Affiliations:** 1 GIMM—Gulbenkian Institute for Molecular Medicine, Avenida Prof. Egas Moniz, 1649-035 Lisboa, Portugal; madalena.pinto@gimm.pt (M.P.); sara.violante@gimm.pt (S.V.); rita.cascao@gimm.pt (R.C.); 2 Department of Neurosurgery, Hospital de Santa Maria, Unidade Local de Saúde de Santa Maria (ULSSM), Avenida Prof. Egas Moniz, 1649-035 Lisboa, Portugal; 3 Clínica Universitária de Neurocirurgia, Faculdade de Medicina da Universidade de Lisboa, Avenida Prof. Egas Moniz, 1649-035 Lisboa, Portugal

**Keywords:** brain metastatic disease, cancer metabolism, metabolic reprogramming, therapeutic targets

## Abstract

The dissemination of malignant cells to the brain is a late-stage complication of cancer, leading to significant morbidity and mortality. Brain metastases (BM) affect 20–30% of cancer patients, primarily originating from lung cancer, breast cancer, and melanoma. Despite advances in molecular-targeted therapies, brain metastatic disease remains incurable, with a poor median survival of ≤12 months if left untreated. The lack of therapeutic efficacy is mainly attributed to the presence of the blood–brain barrier (BBB) and genetic differences between BM and their primary tumors. Previously published data have identified potential driver mutations of BM. However, the mechanisms underlying brain cancer dissemination remain unknown. Recent studies emphasize the pivotal role of metabolic adaptations in supporting the metastatic process, particularly in the nutrient-poor microenvironment characteristic of the brain. Understanding the interplay between metabolism and genetic alterations associated with brain metastatic disease could unveil novel therapeutic targets that are more effective in treating patients. This review focuses on relevant metabolic pathways in cancer, particularly brain cancer dissemination, while also presenting information on current preclinical models of BM, relevant clinical trials, and preclinical studies targeting metabolic reprogramming, providing an overview for advancing therapeutic strategies in BM.

## 1. Introduction

Metastases refer to the dissemination of cancer cells from primary tumors to distant parts of the body, where they colonize and establish secondary tumors. This process is considered a hallmark of malignant cancer, and it is responsible for approximately 90% of cancer-related deaths [1,2]. Despite advances in cancer therapy, current treatments for metastatic disease remain largely ineffective. The severity of metastatic cancer and the lack of effective treatment impose a need for novel therapeutic possibilities.

Throughout the dissemination process, cancer cells encounter multiple environments, each presenting a variety of challenges. Furthermore, the localization of the secondary sites is not a random process, as initially postulated by the “seed and soil” hypothesis [3]. Successful metastases depend on the interaction between the “seed” (the cancer cells) and the “soil” (the microenvironment at the secondary site). Thus, cancer cells must possess specific characteristics that allow them to survive the metastatic process, while the secondary site can either promote or inhibit the growth of metastatic cancer cells.

During brain dissemination, metastatic cancer cells must be able to cross the blood–brain barrier (BBB) and must adapt to the unique brain microenvironment, which differs significantly from the environment of primary tumors [4]. Brain metastases (BM) are the most common brain tumors in adults, and their presence significantly aggravates the clinical outcome of cancer patients [4]. Treatment involves multi-modal therapies, including surgery and radiotherapy, which remain ineffective [5]. Challenges in treatment include metastases heterogeneity, differences between BM and primary tumors, and limited drug delivery due to the BBB, rendering this disease incurable.

In recent years, emerging research has shed light on the intricate interplay between cancer cells and their environment, revealing profound implications for metastatic progression and therapeutic interventions [6]. Metabolic reprogramming emerges as a core hallmark of metastatic cancer cells, enabling them to survive dissemination and thrive in the microenvironment of secondary sites [6,7]. By harnessing the metabolic vulnerabilities inherent to brain metastatic tumors, novel therapeutic opportunities may be explored, enhancing treatment efficacy and improving patient outcomes.

This review explores the current knowledge of metabolic adaptations exhibited by cancer cells, focusing on metastatic cancer cells upon dissemination into the brain, and also presents information on current preclinical models of BM and relevant clinical trials and preclinical studies targeting metabolic reprogramming in the context of BM. We discuss the complex interplay between cellular metabolism and the unique microenvironment of the central nervous system (CNS), providing an overview of possible therapeutic targets that may be used for the future development of innovative treatments.

## 2. Metabolic Pathways in Normal Cells

### 2.1. Glycolysis

**Figure 1:**
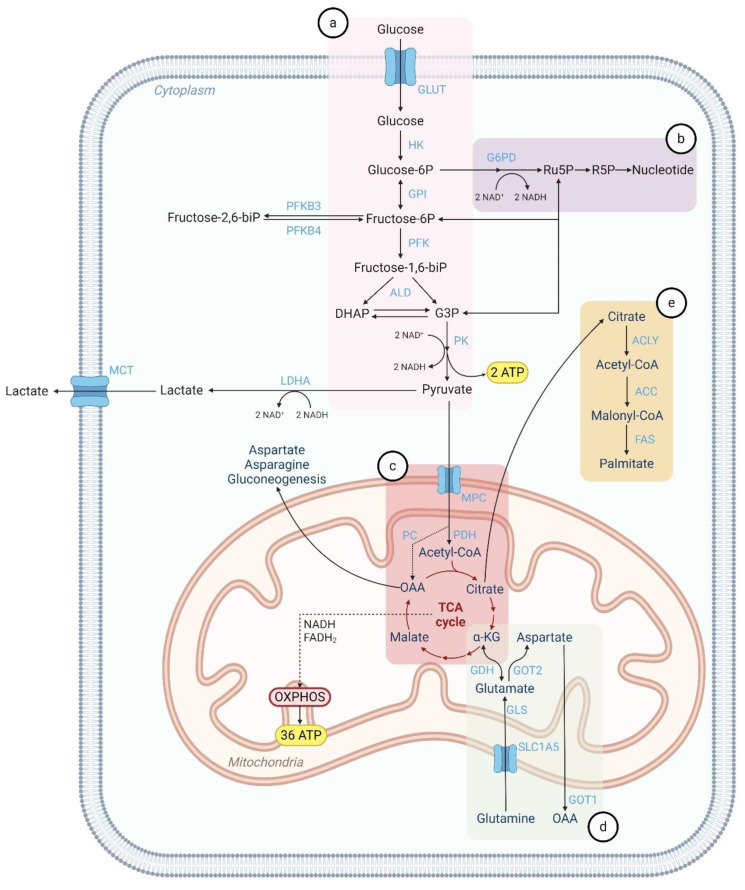
Interplay between the most relevant metabolic pathways in normal cells. Schematic representation of important metabolic reactions in normal cells, including (**a**) glycolysis, (**b**) pentose phosphate pathway, (**c**) tricarboxylic acid cycle, (**d**) glutaminolysis, and (**e**) lipid metabolism. Nutrient transporters and metabolic enzymes are depicted in light blue. GLUT, glucose transporter; HK, hexokinase; GPI, phosphoglucose isomerase; PFK, phosphofructokinase; ALD, fructose bisphosphate aldolase; PK, pyruvate kinase; G6PD, glucose 6-phosphate dehydrogenase; LDHA, lactate dehydrogenase A; MCT, monocarboxylate transporters; ACLY, ATP citrate lyase; ACC, acetyl-CoA carboxylase; FAS, fatty acid synthase; MPC, mitochondrial pyruvate carrier; PDH, pyruvate dehydrogenase; PC, pyruvate carboxylase; GDH, glutamate dehydrogenase; GOT1/2, glutamic-oxaloacetic transaminase 1/2; GLS, enzyme glutaminase. Created in BioRender. Pinto, M. (2025) https://BioRender.com/l94v190.

Glycolysis (Figure 1a) is the initial step in cellular respiration and takes place in the cytoplasm. Glucose enters the cell via glucose transporters (GLUTs) and is first phosphorylated by hexokinase (HK) into glucose-6-phosphate (G6P), which is subsequently converted to fructose-6-phosphate (F6P) by phosphoglucose isomerase (GPI). Phosphofructokinase 1 (PFK) then produces fructose-1,6-bisphosphate (F1,6BP). Fructose-2,6-bisphosphate (F2,6P2) activates PFK by increasing its affinity for F6P. The levels of F2,6P2 are regulated by the enzyme phosphofructokinase B (PFKB). Dihydroxyacetone phosphate (DHAP) and glyceraldehyde 3-phosphate (G3P) are then generated from F1,6BP by fructose bisphosphate aldolase (ALD). In the final step of glycolysis, pyruvate kinase (PK) catalyzes the production of pyruvate from G3P. The overall products of glycolysis are 2 ATP, 2 NADH, and 2 pyruvate molecules. Under aerobic conditions, pyruvate is transported into the mitochondria, where it enters the tricarboxylic acid cycle (TCA cycle or Krebs cycle) and undergoes oxidative phosphorylation (OXPHOS). This process produces carbon dioxide and up to 36 ATP molecules as final products. In contrast, under anaerobic conditions, pyruvate remains in the cytoplasm and is fermented into lactate by the enzyme lactate dehydrogenase A (LDHA). This anaerobic pathway yields 2 ATP and 2 NAD^+^ molecules. The lactate is then transported out of the cell via the monocarboxylate transporters (MCTs).

### 2.2. Pentose Phosphate Pathway (PPP)

The pentose phosphate pathway (PPP) (Figure 1b) is a metabolic pathway that occurs parallel to glycolysis in the cytoplasm of cells. It comprises two interconnected branches: an irreversible oxidative phase and a reversible non-oxidative phase. During the oxidative phase, glucose 6-phosphate dehydrogenase (G6PD) catalyzes the oxidation of G6P, a product of glycolysis, resulting in the formation of ribulose-5-phosphate (Ru5P) and 2 NADH molecules. These products are essential for maintaining cellular redox balance. In the non-oxidative phase, pentose phosphate intermediates undergo rearrangement reactions to produce ribose-5-phosphate (R5P), a precursor for nucleotide synthesis. Alternatively, the non-oxidative phase can produce glycolytic intermediates, such as F6P and G3P. F6P can be reconverted to G6P to sustain the oxidative phase and facilitate additional NADH production. G3P can be metabolized through further steps of glycolysis, ultimately producing pyruvate, NADH, and ATP. The balance between the oxidative and non-oxidative branches of the PPP is regulated by the metabolic state and specific requirements of the cell.

### 2.3. Mitochondrial Metabolism

The final steps of cellular respiration take place in the mitochondria. Pyruvate, produced during glycolysis, is transported into the mitochondria via the mitochondrial pyruvate carrier (MPC). Then, pyruvate enters the TCA cycle (Figure 1c) by two pathways: it is either converted into acetyl-coenzyme A (acetyl-CoA) by pyruvate dehydrogenase (PDH) or transformed into oxaloacetate (OAA) by pyruvate carboxylase (PC). The cycle is initiated with the condensation of OAA with acetyl-CoA, generating citrate. As the cycle progresses, various intermediate products are produced, including isocitrate, α-ketoglutarate (αKG), succinyl-CoA, succinate, fumarate, and malate. These intermediates can be redirected from the cycle to fuel other metabolic pathways, such as gluconeogenesis (generation of glucose from non-carbohydrate substrates), lipid synthesis, and amino acid metabolism. For instance, αKG can be converted into glutamate, a key precursor for amino acid and nucleotide synthesis (Figure 1d). Similarly, when citrate is exported to the cytoplasm, it is converted into acetyl-CoA by ATP citrate lyase (ACLY), which is vital for lipid metabolism (Figure 1e). Malate can also exit the mitochondria and be converted back into pyruvate. OAA contributes to the synthesis of non-essential amino acids, such as aspartate and ultimately asparagine, essential for protein and nucleotide biosynthesis. Additionally, OAA can be converted into phosphoenolpyruvate, a precursor for gluconeogenesis. NADH and FADH_2_ are also generated during the TCA cycle. These are carriers of high-energy electrons, which are transferred to the electron transport chain (ETC) located in the inner mitochondrial membrane. Through OXPHOS, these electrons drive the phosphorylation of ADP to ATP, yielding up to 36 ATP molecules.

### 2.4. Glutaminolysis

Glutaminolysis (Figure 1d) is a mitochondrial pathway that generates energy through the degradation of glutamine, the most abundant amino acid in the body. Glutamine is transported into mitochondria via the SLC1A5 transporter and catalyzed into glutamate by the enzyme glutaminase (GLS), releasing nitrogen in the form of ammonia. Glutamate can then be further metabolized by glutamate dehydrogenase (GDH) into αKG, which enters the TCA cycle to produce energy. Alternatively, mitochondrial aspartate aminotransferase GOT2, also known as glutamic-oxaloacetic transaminase 2, can generate aspartate and αKG from glutamate and OAA. In contrast, the cytosolic form of the enzyme (GOT1) catalyzes the reverse reaction.

### 2.5. Lipid Metabolism

Acetyl-CoA serves as the precursor for de novo fatty acid synthesis (Figure 1e) in the cytoplasm. Since acetyl-CoA cannot cross the mitochondrial membrane, it relies on the production of citrate during the TCA cycle. Citrate is transported to the cytoplasm via the mitochondrial citrate/isocitrate carrier (CIC) and is enzymatically cleaved by ACLY, yielding cytoplasmic OAA and acetyl-CoA. OAA is subsequently reduced to malate, which either re-enters the mitochondria or remains in the cytoplasm to undergo oxidation into pyruvate. Acetyl-CoA is then directed towards fatty acid and cholesterol synthesis. Fatty acid synthesis begins with the conversion of acetyl-CoA to malonyl-CoA, a reaction catalyzed by acetyl-CoA carboxylase (ACC). Acetyl-CoA and malonyl-CoA further generate acetyl-ACP and malonyl-ACP by acetyl transacylase and malonyl transacylase, respectively. The multienzyme complex fatty acid synthase (FAS) then catalyzes a series of reactions that convert acetyl-ACP and malonyl-ACP into palmitate, a 16-carbon saturated fatty acid. Palmitate can then undergo two additional processes: elongation, catalyzed by the fatty acid elongase (FAE), to generate longer fatty acids, or desaturation, catalyzed by stearoyl-coenzyme A desaturase 1 (SCD1), to produce monounsaturated fatty acids (MUFAs).

## 3. Metabolic Reprogramming in Cancer Cells

Under normal conditions, cells predominantly rely on mitochondrial OXPHOS as the primary energy source for cellular processes, growth, and survival, switching to fermentation only when oxygen supply is limited. Cancer cells, however, undergo rapid and uncontrolled proliferation, a process highly dependent on the increased uptake of nutrients, biosynthesis of building blocks for cellular components, and acquisition of energy to support abnormal growth. In the 1920s, Warburg observed that, unlike normal cells, cancer cells preferentially convert glucose to lactate even in the presence of oxygen [8]. This is the best-described metabolic reprogramming in cancer cells, named the “Warburg effect” or “aerobic glycolysis”, which represents a metabolic shift from the OXPHOS pathway to anaerobic glycolysis, despite the presence of oxygen [9]. This shift is regulated by oncogenes such as *MYC*, *HIF1α* (hypoxia-inducible factor 1 subunit alpha), and *PIK3Cα* (phosphatidy linositol-4,5-bisphosphate 3-kinase catalytic subunit alpha) [10,11]. Although anaerobic glycolysis is energetically less efficient than OXPHOS in terms of ATP production, it generates intermediates, including carbon and NADH molecules, which are essential for the biosynthesis of macromolecules required for rapid cell growth and proliferation [9]. Additionally, anaerobic glycolysis generates ATP at a faster rate, meeting the high energy demands of rapidly dividing cancer cells. By relying on glycolysis, cancer cells also avoid the excessive production of reactive oxygen species (ROS) associated with OXPHOS, which could cause cellular damage in highly proliferative states. Furthermore, anaerobic glycolysis enables cancer cells to survive in hypoxic environments often found in the tumor microenvironment (TME). Nevertheless, metabolic reprogramming in cancer goes beyond the “Warburg effect”, encompassing alterations in other metabolic pathways. For instance, increased glutaminolysis plays a role in cellular proliferation through protein and nucleotide synthesis, which are essential for the rapid growth and division of cancer cells [12]. Notably, glutamine is a major substrate for the TCA cycle, contributing to ATP generation and serving as an alternative energy source [12]. Enhanced lipid uptake and de novo lipid biosynthesis also support rapid cancer cell growth and proliferation in tumors by fueling cell membrane formation, facilitating intercellular signaling, and acting as an additional energy source [13]. As research in cancer biology advances, the understanding of the unique metabolic features of cancer cells remains crucial and an evolving area of study.

## 4. Metabolic Adaptation in Brain Metastatic Disease

**Figure 2:**
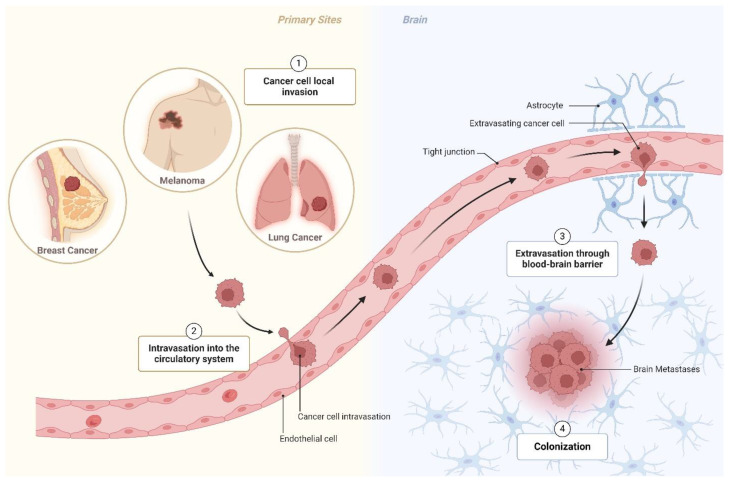
Brain metastatic process. Metastatic brain cancer predominantly originates from lung, breast, and melanoma tumors. Metastasis is a complex multistep process that comprises (1) local tissue invasion into adjacent tissues, (2) intravasation into the circulatory system and survival in the bloodstream, (3) extravasation to brain pre-metastatic sites, and (4) colonization and proliferation in the brain metastatic site. Created in BioRender. Pinto, M. (2025) https://BioRender.com/l94v190.

Brain dissemination is a late-stage complication in cancer patients and is a leading cause of morbidity and mortality. If left untreated, the overall patient survival is ≤12 months following diagnosis [14]. It is estimated that 10–40% of cancer patients develop BM during the progression of their disease, although the actual incidence might be higher [15]. This increased incidence is likely attributed to advances in BM diagnostic methods and improvements in primary tumors’ treatment, which extend patient survival. BM can arise from any type of cancer, but the most common primary tumors associated with brain dissemination are lung cancer (43% to 45% of cases), breast cancer (15% to 18%), and melanoma (16% to 20%) [16,17]. As cancer cells disseminate, they encounter diverse microenvironments and must adapt to secondary sites (Figure 2). The TME in primary tumors differs significantly from that of the CNS. Cancer cells can metastasize to the brain parenchyma, leptomeninges, or cerebrospinal fluid (CSF), each compartment harboring a unique metabolic milieu [5]. Furthermore, the BBB tightly limits the transport of nutrients and cells into the brain [4]. Specific BBB transport systems facilitate the passage of essential nutrients and molecules, such as glucose, amino acids, and nucleotides [4]. Another hallmark of the CNS is hypoxia, which imposes additional stress on disseminated cancer cells. These distinct conditions collectively challenge brain metastatic cancer cells, shaping their metabolism. Metabolic reprogramming is thus essential for metastatic cancer cells to adapt to the brain microenvironment and survive. The three main pathways affected are glycolysis, glutaminolysis, and lipid metabolism (Figure 1).

### 4.1. Glycolysis and TCA Cycle

Glucose is an abundant metabolite in the brain and is used as the main energy substrate. Unlike the prevalent anaerobic glycolysis observed in many cancer cells within oxygen-deprived TMEs, brain metastatic cancer cells might exhibit a metabolic shift favoring the OXPHOS pathway (Figure 1). Studies reported an increased expression of enzymes involved in glycolysis, the TCA cycle, and the OXPHOS pathways in brain metastatic breast cancer cells [18,19]. In response to oxidative stress, these cells also upregulated alternative pathways, such as the PPP, to mitigate the generation of ROS [18]. Variations in genes encoding OXPHOS-related proteins were also correlated with brain metastatic breast cancer [20]. Using a sensitive deep-sequencing approach, researchers identified structural and functional variations in mitochondrial DNA-encoded OXPHOS proteins derived from blood samples of breast cancer patients who later developed BM [20]. Similarly, gene expression analysis by RNA sequencing, direct metabolite profiling, and in vivo ^13^C-glucose tracing of melanoma BM samples revealed increased OXPHOS activity in these metastases compared to patient-matched extracranial metastases and primary melanoma tumors [21]. Concordantly, gene expression profiling of BM and their patient-matched primary or extracranial metastatic tissues from lung, breast, and renal cell carcinomas revealed an upregulation of OXPHOS in BM [22]. Furthermore, another RNA-sequencing study found a strong association between the expression of specific OXPHOS genes and the risk of BM in non-small cell lung cancer (NSCLC), relating an elevated OXPHOS pathway activity to a higher BM risk [23]. Thus, metastatic brain cancer appears to involve both increased glycolysis and OXPHOS, diverging from the classical “Warburg effect”. This evidence suggests that BM cells reprogram their metabolic pathways to adapt to the brain TME, using glucose oxidation via the OXPHOS pathway as a primary energy source. However, it remains unclear whether the metabolic profile varies based on the primary tumor’s origin or specific microenvironmental conditions within the brain.

### 4.2. Glutaminolysis

Glutamine is an important amino acid in the brain, functioning as both a carbon and nitrogen source and a precursor for the synthesis of TCA cycle intermediates, which are essential for energy production and macromolecule biosynthesis. Although glucose is considered the primary energy substrate in the brain, a study demonstrated that brain metastatic breast cancer cells continued to proliferate and survive even in glucose-deprived conditions, unlike their parental cell line [24]. This survival was attributed to the activation of alternative metabolic pathways, including gluconeogenesis and increased glutaminolysis (Figure 1). Also, melanoma cells that metastasize to the brain undergo transcriptional changes that result in a brain-specific phenotype adapted to this environment [25]. Researchers observed the upregulation of genes involved in glutamate receptor signaling in melanoma BM samples [25]. Importantly, the inhibition of glutamate receptors was shown to reduce BM growth in vitro. These findings suggest that glucose might not be the sole primary energy source for BM cells. Instead, they undergo metabolic reprogramming to utilize alternative energy sources, such as glutamine, enabling their proliferation and survival in the brain TME.

### 4.3. Lipid Metabolism

Lipids are crucial for brain cellular functions, comprising approximately 50% of the brain’s dry weight [26]. Lipid metabolism plays an important role in BM (Figure 1). Previous research unveiled a distinct lipid signature in brain metastatic breast cancer cells, characterized by elevated cholesterol and membrane lipid levels [27]. This study also described that sterol regulatory element-binding protein (SREBP1) was highly correlated with BM [27]. Moreover, SREBP1 was found to be essential for the growth of BM cells in vitro, in contrast to breast cancer cell lines with low or no brain metastatic potential [27]. SREBP1 is a transcription factor that mediates de novo lipid biosynthesis, regulating the expression of ACYL, ACC, FAS, and SCD1 [28]. Recently, Li et al. explored the mechanisms of lipid metabolic reprogramming in breast cancer BM and identified that the retinoic acid receptor responder 2 (RARRES2) gene was significantly downregulated and was associated with enhanced lipid synthesis and fatty acid metabolism signatures [29]. This alteration activates the phosphatase and tensin homolog (PTEN)/mammalian target of rapamycin (mTOR)/SREBP1 signaling pathway, which is also associated with lipid biosynthesis [29]. Concordantly, Ferraro et al. demonstrated that brain metastatic breast cancer cells rely on de novo lipid biosynthesis to sustain proliferation and survival in the brain TME [30]. Another study reported a correlation between a higher incidence of BM and increased expression of fatty acid binding protein 7 (*FABP7*) in breast metastatic cancer cells [31]. This protein was found to promote lipid droplet storage and maintain a glycolytic phenotype in these cells [31]. Additionally, melanoma cancer cells with brain tropism exploit the lipid-rich brain environment to support metastatic growth through disrupted signaling [32]. Astrocytes, with their high polyunsaturated fatty acid content, activate the proliferator-activated receptor γ (PPARγ) pathway in the surrounding cancer cells, enhancing their proliferation [32]. Collectively, these studies highlight the critical role of lipid metabolism, specifically of distinct lipid signatures and the regulation of key lipid-related factors, in enabling metastatic cancer cells to adapt to the brain TME.

### 4.4. Metabolic Interactions Between the Brain Microenvironment and BM

The dissemination of cancer cells to the brain is influenced by intrinsic metabolic reprogramming and the adaptation of these cells to the brain microenvironment, but it is also regulated by this environment. When cancer cells reach the brain, they encounter a complex microenvironment that differs from the primary site, including a range of unique cellular constituents such as neurons and astrocytes. Thus, the metabolic milieu also differs from the primary site. Neurons and astrocytes are two key components of the brain microenvironment that maintain neuronal function and support brain metabolism. BM cells form an interconnected network with these brain cells by expressing neurotransmitter receptors, such as gamma-aminobutyric acid receptor type-A subunit B1 (GABRB1) and *N*-methyl-d-aspartate receptor (NMDAR) [33,34]. Gamma-aminobutyric acid (GABA) released from neurons is taken up by cancer cells to generate succinate and feed the TCA cycle [33]. The neurotransmitter glutamate binds and activates NMDAR in cancer cells, allowing for calcium influx and triggering downstream signaling pathways [34]. BM cells also express synapse-associated proteins, including synaptosomal-associated protein 25 (SNAP25) and postsynaptic density protein 95 (PSD-95) [33]. This enables BM cells to integrate neuronal signals and activate downstream signaling pathways to meet their metabolic demands. Additionally, BM cells form compact spheroid structures that segregate stromal cells to the periphery, facilitating the interaction with neurons and astrocytes, promoting metabolic support [35]. Tumor cells can also activate reactive astrocytes that promote metabolic adaptation, supporting tumor cells’ survival. BM cells establish gap junctions with astrocytes, which supply cancer cells with essential metabolites, including glucose, lactate, and glutamate [36]. In turn, cyclic GMP-AMP (cGAMP) is transferred from BM cells to astrocytes via the gap junctions, which activate the PPP in astrocytes [36]. This activation leads to an increased production of nucleotides and antioxidants while increasing glutamate and GABA availability [36]. These metabolites are subsequently shuttled back to tumor cells, enhancing their survival and proliferation under hypoxic and nutrient-deprived conditions [36]. Altogether, these complex metabolic interactions between BM cells and the brain microenvironment sustain tumor growth in a nutrient-restricted niche during the metastatic process in the brain.

## 5. Regulation of Cancer Metabolism by Oncogenes and Tumor Suppressors

**Figure 3:**
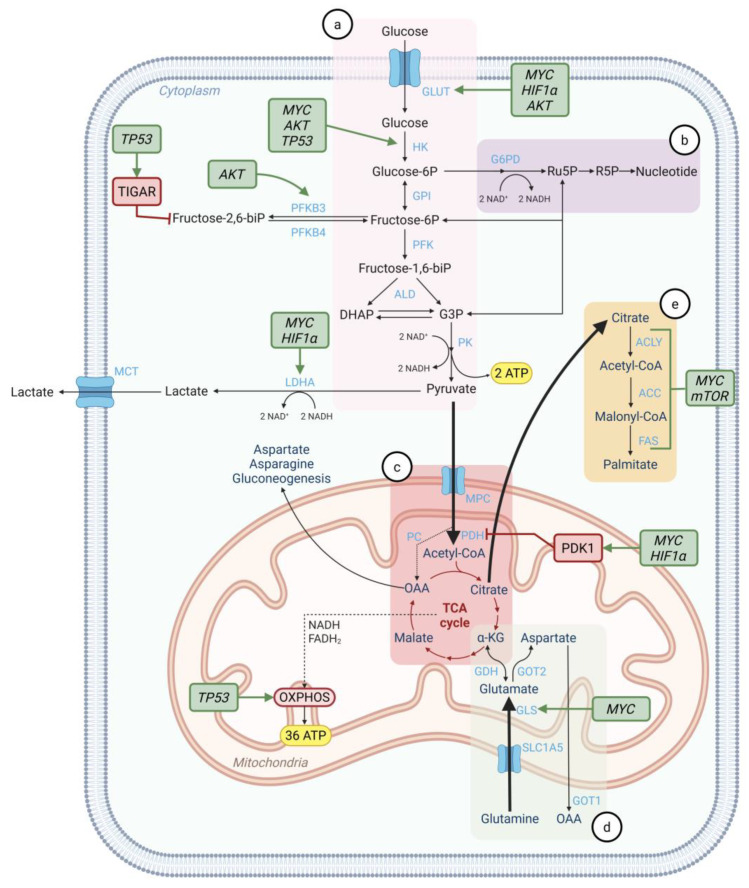
Interplay between the most relevant metabolic pathways, oncogenes, and tumor suppressors. Schematic representation of important metabolic reactions in cancer cells, including (**a**) glycolysis, (**b**) the pentose phosphate pathway, (**c**) the TCA cycle, (**d**) glutaminolysis, and (**e**) lipid metabolism. In BM, not only is glycolysis increased, but also the OXPHOS pathway, glutaminolysis, and lipid metabolism seem to be upregulated. Relevant oncogenes and tumor suppressors, including *MYC*, *HIF1α*, *mTOR*, *AKT*, and *TP53*, are depicted in green when stimulating or in red when repressing mediators. Nutrient transporters and metabolic enzymes are depicted in light blue. GLUT, glucose transporter; HK, hexokinase; GPI, phosphoglucose isomerase; PFK, phosphofructokinase; ALD, fructose bisphosphate aldolase; PK, pyruvate kinase; G6PD, glucose 6-phosphate dehydrogenase; LDHA, lactate dehydrogenase A; MCT, monocarboxylate transporters; ACLY, ATP citrate lyase; ACC, acetyl-CoA carboxylase; FAS, fatty acid synthase; MPC, mitochondrial pyruvate carrier; PDH, pyruvate dehydrogenase; PC, pyruvate carboxylase; GDH, glutamate dehydrogenase; GOT1/2, glutamic-oxaloacetic transaminase 1/2; GLS, enzyme glutaminase. Created in BioRender. Pinto, M. (2025) https://BioRender.com/l94v190.

Metabolic reprogramming in metastatic cancer cells is tightly regulated by oncogenic and tumor suppressor gene mutations (Figure 3).

***MYC*** is a proto-oncogene frequently associated with cancer development that regulates various metabolic pathways, including glycolysis, glutaminolysis, and lipid metabolism [37]. In response to the TME, *MYC* can activate aerobic glycolysis by increasing the expression of GLUT1, HK, pyruvate dehydrogenase kinase 1 (PDK1), and LDHA [37]. Additionally, MYC was shown to upregulate transport proteins and enzymes related to glutaminolysis, including GLS and SLC1A5, thereby enhancing the conversion of glutamine to glutamate and fueling the TCA cycle and OXPHOS [38,39]. More recently, *MYC* was reported to augment lipid biosynthesis by activating the expression of ACLY, ACC, and FAS [37,40]. In BM, *MYC* is upregulated, promoting macrophage recruitment and the formation of gap junctions between metastatic cells and astrocytes, which support invasive metastatic growth [41].

***HIF1α*** encodes the alpha subunit of hypoxia-inducible factor 1 (*HIF1*), a transcription factor pivotal in regulating cellular response to hypoxia. Its expression is modulated by oxygen availability. Under normoxic conditions, the tumor suppressor von Hippel–Lindau (*VHL*) targets *HIF1α* for ubiquitination, preventing the activation of hypoxia-responsive genes [42]. However, *HIF1α* can be activated in normoxic environments due to *VHL* mutations or the accumulation of metabolites such as fumarate and succinate from the TCA cycle [43]. In hypoxic conditions, which are common in highly proliferative tumors, *HIF1α* promotes the expression of hypoxia-responsive genes involved in glycolysis, angiogenesis, and metastasis, enabling cancer cells to adapt to low oxygen levels and sustain their growth [44]. *HIF1α* upregulates the transcription of glycolytic genes encoding GLUT1 (to increase glucose uptake), several glycolytic enzymes, and LDHA (to stimulate the conversion of pyruvate to lactate) [45]. It also activates PDK1, which inhibits PDH, thereby reducing the rate of OXPHOS [45]. In BM, *HIF1α* expression is upregulated compared to matched primary tumors [46]. Moreover, suppression of *HIF1α* signaling in circulating tumor cells decreased tumorigenesis in the brain [46].

***PIK3Cα*** encodes the catalytic subunit alpha of phosphoinositide 3-kinase (*PI3K*). This gene is frequently mutated in various cancers, leading to the activation of the PI3K/AKT/mTOR signaling pathway, which promotes cell growth, proliferation, and metabolism [47]. Mutations in tumor suppressor genes, such as *PTEN*, can also activate this pathway. Downstream of *PIK3*, *AKT* stimulates glycolysis by increasing the expression of GLUTs and glycolytic enzymes, including HK and PFKFB3 [47]. Additionally, *AKT* indirectly activates *mTOR*, which promotes lipid and protein synthesis, while also activating transcription factors, including *HIF1α* [47,48]. In BM, the PI3K pathway activation was associated with enhanced OXPHOS when compared to patient-matched primary or extracranial metastatic tissues [22].

***TP53*** is a tumor suppressor gene that impacts glycolysis and OXPHOS [49]. While it activates the expression of HK, the enzyme catalyzing the first step of glycolysis, *TP53* can also inhibit this pathway by upregulating the expression of *TP53*-induced glycolysis and apoptosis regulator (*TIGAR*), which reduces the levels of F2,6BP, a key glycolysis intermediate [50]. The decrease in F2,6BP can inhibit glycolysis as this metabolite serves as an allosteric activator of phosphofructokinase. Furthermore, *TP53* increases the expression of *PTEN*, which suppresses the *PI3K* pathway, therefore diminishing glycolytic activity [51]. *TP53* also modulates the shift of glycolysis and the OXPHOS pathways via synthesis of cytochrome *c* oxidase (SCO2), which maintains the assembly of the cytochrome *c* oxidase (COX) complex and enhances mitochondrial respiration in normal conditions [20]. Interestingly, studies described an increased frequency of *TP53* mutations in brain metastatic breast cancer [52]. Moreover, cells metastasizing to the brain exhibit clonal selection for *TP53* mutations compared to matched primary tumors.

Despite the association between BM and these oncogenes and tumor suppressors, there are currently no studies investigating their role in the metabolic adaptation of disseminated cancer cells to the brain TME.

## 6. Current Preclinical Models of Brain Metastasis to Test New Therapies

The metastatic cascade (depicted in Figure 2) is a complex multistep process that comprises local tissue invasion, intravasation into the circulatory system, extravasation to brain pre-metastatic sites, and colonization of the metastatic site. Within the brain, metastatic cancer cells encounter a distinct microenvironment, requiring their adaptation to survive [53]. Additionally, the brain is shielded by the BBB, a highly selective semi-permeable barrier between the blood and the CNS. To understand the molecular mechanisms underlying the metabolic reprogramming in brain metastatic cancer cells, it is crucial to use preclinical models that rigorously reflect the complexity of the brain metastatic process. These models also serve as important platforms to test new therapies. Current preclinical models include in vitro and in vivo models, as described below.

### 6.1. In Vitro Models

In a **two-dimensional (2D) cell culture monolayer**, cancer cells are grown on the flat surface of a culture flask or petri dish. This model allows for direct cell–cell interactions and the study of processes like migration, invasion, proliferation, and apoptosis. Also, it ensures a controlled and uniform distribution of nutrients, oxygen, and tested therapeutics. It is a reproducible model and allows for high-throughput screenings, such as drug screenings. Nonetheless, 2D cultures lack the TME complexity and do not allow the study of all the metastasis cascade steps.

The **BBB transwell model** is a two-chamber cell culture system that consists of a permeable membrane insert, an upper chamber that mimics the vascular system, and a lower chamber that mimics the neurovascular unit [54]. Endothelial cells are seeded on the upper chamber, forming a tight monolayer that mimics the BBB endothelium due to the tight junction proteins, which ensure selective permeability for various substances [54]. Cancer cells are also seeded in the upper chamber, while astrocytes, pericytes, and other cerebral cells are added to the lower chamber to mimic the brain environment [55]. This model can be used to assess the transmigration of cancer cells and the transport of therapeutic drugs across the BBB. In recent years, the BBB transwell model has been increasingly employed in multiple BM studies, offering a controlled and reproducible platform [56,57,58,59]. Despite the increased complexity of this model, it lacks complete brain tissue 3D architecture, blood flow, and hemodynamics.

**Three-dimensional (3D) brain organoids** are self-organizing 3D structures formed by stem cells from healthy or tumor tissue samples that recapitulate the brain architecture, microenvironment, and functional properties [60]. The co-culture of cancer cells with brain organoids leads to the adhesion, migration, and proliferation of these cells into the brain organoids, mimicking the brain metastatic process [55]. This model offers a valuable platform for drug screening and the evaluation of personalized therapeutic strategies. Organoid models have become increasingly prominent in recent BM research, reflecting their growing relevance in modeling tumor–brain interactions [61,62,63,64]. However, establishing organoid culture conditions can be challenging, as the process is time-consuming, technically demanding, and associated with high costs [65]. **Microfluidic BBB models** are chip-based devices that closely mimic the structure, function, and dynamics of the BBB. The model overcomes the limitation of the previously presented static in vitro models, since it incorporates fluid flow, shear stress, and cell–cell interactions. Brain endothelial cells are cultured within microchannels, often alongside astrocytes, pericytes, or neurons, to recreate the neurovascular unit. The cell culture medium is pumped through these channels, replicating blood flow. The microfluidic BBB model has gained importance as a tool for studying BM due to its physiological relevance, enabling the investigation of cancer cell transmigration and drug permeability [66,67]. However, despite their advantages, these models require specialized equipment and expertise and are less suited for high-throughput screening compared to simpler systems.

### 6.2. In Vivo Models

**Syngeneic models** are transplantation models obtained by injecting a recipient mouse of a specific genetic background with cell lines previously established through the isolation of cancer cells from a mouse with the same genetic background [55]. This system prevents immune rejection, enabling tumor growth within an intact and fully functional immune system. This model is thus particularly relevant for studying the process of metastasis or immune-related responses of cancer cells. This model is also suited for testing therapeutics [55].

**Patient-derived xenografts (PDXs)** consist of transplanting a small piece of a human tumor sample, organoid, or patient-derived cells (PDCs) into an immune-deficient mouse [55]. The site of implantation can vary depending on each study’s objectives, but in BM research, the most common are subcutaneous, intracardiac, tail vein injection, or intracranial models [55,65]. For example, Faria et al. generated PDCs from BM biopsy samples grown in subcutaneous mice PDXs and used them to generate intracardiac PDXs [68]. With these approaches, mice developed metastases, including BM, and recapitulated the clinicopathological features of the donor patients [68]. These models offer a significant advantage in BM research by preserving the genetic, histological, and molecular characteristics of donor patient tumors. Also, these models more accurately reflect therapeutic responses compared to traditional cell lines or syngeneic models. PDXs also support personalized medicine approaches, serving as patient “avatars” for evaluating potential innovative treatments.

## 7. Targeting Metabolic Mediators for Future BM Therapeutics

Appropriate treatments for BM depend on the number, size, and location of these lesions as well as on the type of primary tumor [69]. Currently, standard-of-care treatment includes surgical resection and radiotherapy, which remain largely ineffective. Surgery is only feasible for a small number of metastases and is restricted to accessible, non-eloquent areas of the brain [70,71]. Radiotherapy, which can target either the entire brain (whole brain radiation therapy, WBRT) or specific areas (stereotactic radiotherapy), is often used in combination with surgery [71]. However, it is associated with the risk of radiation-induced toxicity and neurologic deterioration [69]. Systemic therapies for the primary tumor have limited efficacy in treating BM and are not routinely used for these patients [71]. This lack of efficacy is due to the poor ability of chemotherapeutic agents to cross the BBB and genetic differences between primary tumors and BM. The BBB is a highly selective semi-permeable barrier between the blood and the CNS, representing a significant challenge for drug delivery to the brain. Additionally, genetic differences between primary tumors and BM result in variations in molecular profiles, which may explain the ineffectiveness of drugs targeting primary tumors in treating BM. Given the complexities involved in treating brain metastatic disease, exploring alternative therapeutic possibilities is crucial. Targeting metabolic reprogramming in BM stands as a promising strategy for future therapeutic intervention. Nevertheless, since normal brain cells use identical metabolic pathways as brain metastatic cancer cells, the potential for unintentional toxic effects from metabolic-targeted treatments must be carefully considered.

Studies using treatments targeting metabolic pathways in BM showed promising results (Table 1). In a phase 1 clinical trial (NCT01111097), the oral administration of dichloroacetate (DCA) demonstrated safety, tolerability, and practicality for long-term use in patients with BM [72]. At the molecular level, DCA is able to cross the BBB and inhibit PDK [73]. By inhibiting PDK, DCA indirectly activates PDH, leading to the conversion of pyruvate into acetyl-CoA and promoting aerobic respiration. This metabolic shift redirects cells from glycolysis to the OXPHOS pathway [73]. This study included fifteen adults with recurrent malignant brain tumors, which comprised thirteen patients with malignant gliomas (a type of primary brain tumor) and two patients with parenchymal metastases [72]. The two patients with BM had a primary diagnosis of papillary serous adenocarcinoma of the uterus and adenocarcinoma of the lung. As a phase 1 clinical trial, this study did not provide data about treatment efficacy. However, it was noted that DCA therapy was associated with clinical and radiographic evidence of disease stabilization. This is in agreement with previous preclinical studies that reported DCA activity in the brain parenchyma and its anti-tumor effects on brain tumors [73]. Although these are promising results, DCA has not advanced in clinical trials, possibly due to its significant toxicity at effective doses, short-lived metabolic effects in vivo, and lack of compelling clinical evidence [74]. Brain dissemination has also been linked to lipid metabolism. TVB-3166, an FAS inhibitor, successfully suppressed lipid synthesis and palmitate levels in in vitro cell cultures of a breast tumor cell line. It also increased cancer cell death in brain tumor-derived slices [30]. Currently, TVB-2640, a related FAS inhibitor, is undergoing phase 2 clinical trials (NCT03179904) for treating metastatic breast cancer patients, though no results have been reported so far. Interestingly, in BM cell lines, TVB-2640 was shown to upregulate fatty acid synthesis as a compensatory response to FAS inhibition and to downregulate cell expression of cycle progression genes [75]. Another FAS inhibitor, BI99179, showed preclinical efficacy by diminishing lipid synthesis and palmitate levels in vitro and by reducing brain tumor growth in mouse xenografts generated by the intracranial implantation of a breast cancer cell line [30].

More comprehensive studies are essential to fully characterize the metabolic profile of brain metastatic cancer cells and to develop effective therapies targeting metabolic pathways in the context of BM.

## 8. Discussion and Future Directions

Understanding the molecular mechanisms and the metabolic adaptations of cancer cells to the brain environment provides crucial insights for developing new and more effective therapeutic strategies to treat patients with BM. The metabolic reprogramming observed in metastatic cancer cells underscores the complexity of cancer adaptation to the brain. While the “Warburg effect” is a well-known phenomenon in cancer, the metabolic profile of brain metastatic cancer cells may diverge from this classical model. Studies have demonstrated an increase in OXPHOS alongside glycolysis, indicating a metabolic shift that favors the use of alternative energy substrates. This metabolic plasticity suggests that targeting glycolysis alone may be insufficient to control the growth of BM. Glutaminolysis has emerged as another key pathway exploited by brain metastatic cancer cells, offering a promising avenue for therapeutic intervention. Likewise, lipid metabolism plays a pivotal role in enabling metastatic cancer cells to adapt to the brain TME. Distinct lipid signatures and the dysregulation of lipid-related factors highlight the therapeutic potential of targeting this pathway in BM.

Importantly, agents inhibiting lipid metabolism have already shown efficacy in preventing brain metastatic progression in preclinical studies. Therapeutic interventions targeting the lipid metabolism pathway are currently being evaluated in phase 1 and 2 clinical trials for treating BM. Data from these trials are essential to better predict the efficacy of these approaches in human disease. No compound has yet advanced to phase 3 or 4 clinical trials, highlighting the challenges in translating preclinical findings into clinically effective treatments. The study of metabolic reprogramming in BM requires models that rigorously reflect the complexity of the brain metastatic process. Although current models do not entirely recapitulate this complex process, emerging innovative platforms offer promising avenues for future research and to test personalized therapies, such as digital twins of patients. A digital twin is a virtual replica of a biological system, created using multi-modal data, such as imaging, genomic, and clinical information [76,77,78]. These models enable a range of applications, including tumor growth modeling, personalized treatment simulations, and in silico clinical trials, making them a powerful asset in the evolution of precision oncology in BM [76,77,78]. Future research should focus on revealing the intricate crosstalk between metabolic pathways and signaling networks in brain metastatic disease to better identify novel metabolic targets. Overcoming the challenges posed by BBB and improving drug delivery are crucial steps towards developing effective therapeutic strategies. Ultimately, metabolic-targeted approaches when used in combination therapy have the potential to significantly improve the prognosis of patients with brain metastatic disease.

## Figures and Tables

**Table 1:** Summary of clinical trials and preclinical studies using treatments targeting metabolic pathways in brain metastatic disease.

Study	Drug Name	Mechanism of Action	Metabolic Pathway Affected	Phase of Clinical Trial (Trial ID/Status)	Key Findings
Dunbar, E.M. et al. (2014) [72]	Dichloroacetate (DCA)	PDK inhibitor	Glycolysis/TCA cycle	Phase 1 (NCT01111097/Completed)	Safe, tolerable, and feasible for chronic administration in adults.
Ferraro, G. et al. (2021) [30]	TVB-3166	Fatty acid synthase (FAS)inhibitor	Lipid metabolism	Preclinical study (N/A)	Increased cancer cell death ex vivo.
Serhan, H. et al. (2024) [75]	TVB-2640	Fatty acid synthase (FAS) inhibitor	Lipid metabolism	Phase 2 (NCT03179904/Ongoing)	Data are still not available.
Ferraro, G. et al. (2021) [30]	BI99179	Fatty acid synthase (FAS) inhibitor	Lipid metabolism	Preclinical study (N/A)	Decreased brain tumor growth in vivo.

## Data Availability

No new data were created or analyzed in this work.
